# Polyamine treatments: an effective strategy to improve crop yield and fruit colour and to preserve quality of ‘Mollar de Elche’ pomegranate fruit during storage at chilling temperatures

**DOI:** 10.1002/jsfa.70542

**Published:** 2026-02-25

**Authors:** Jenifer Puente‐Moreno, Fernando Garrido‐Auñón, María E García‐Pastor, Fabián Guillén, Salvador Castillo, Daniel Valero, María Serrano

**Affiliations:** ^1^ Department of Applied Biology, CIAGRO University Miguel Hernández Orihuela (Alicante) Spain; ^2^ Department of Food Technology, CIAGRO University Miguel Hernández Orihuela (Alicante) Spain

**Keywords:** anthocyanins, chilling injury, phenolics, *Punica granatum* L., putrescine, spermidine

## Abstract

**BACKGROUND:**

Climate change in Spain is altering the optimal conditions for producing high‐quality ‘Mollar de Elche’ pomegranate fruit, due to reduced colour development in husk and arils. In addition, the pomegranate requires storage above 7–10 °C, depending on cultivar, to preserve its quality because it is susceptible to chilling injury (CI) damage at lower temperatures.

**RESULTS:**

In this study, the effect of preharvest application of 0.01 and 0.1 mmol L^−1^ spermidine (SPD) and putrescine (PUT) on crop yield and fruit quality at harvest was evaluated in two consecutive years, 2022 and 2023, and during cold storage at chilling temperature in fruit for the 2023 trial. Results showed that SPD and PUT, at 0.01 and 0.1 mmol L^−1^, significantly increased crop yield and improved fruit quality parameters at harvest in the experiments carried out in 2022 and 2023. The application of polyamines (PAs) effectively alleviated CI after 30, 60 and 90 days of storage at 2 °C (85–90% RH) plus 2 days at 20 °C (55–60% RH) by reducing electrolyte leakage, malondialdehyde accumulation and peel browning. PAs also preserved fruit firmness and titratable acidity and decreased respiration rate and weight loss. In addition, the ripening index was significantly lower in PA‐treated fruit compared to controls during the whole storage period. Finally, PAs increased total phenolic content in arils, especially anthocyanins, which intensified aril red colour. Notably, SPD at 0.01 mmol L^−1^ was the most effective treatment in improving crop yield, red colour of husk and arils and anthocyanin content in arils with strong effects also in reducing CI symptoms.

**CONCLUSION:**

The findings demonstrate the potential of PAs, especially SPD at 0.01 mmol L^−1^, as an effective and sustainable tool to improve crop yield, mitigate CI and preserve the quality of ‘Mollar de Elche’ pomegranate fruit during cold storage. Thus, PA treatments would lead to an increase in growers' income and health benefits for consumers, due to the enhanced bioactive compound content. © 2026 The Author(s). *Journal of the Science of Food and Agriculture* published by John Wiley & Sons Ltd on behalf of Society of Chemical Industry.

## INTRODUCTION

‘Mollar de Elche’ pomegranate, a cultivar of the species *Punica granatum* L., was the first cultivar to be recognised by the Protected Designation of Origin, a recognition that highlights the close link between its physicochemical quality and the geographical, climatic and human characteristics of its production area, located in the south of the Community of Valencia (Spain). Pomegranate fruit is a rich source of polyphenols, bioactive compounds found in different parts of the fruit which are responsible for its effects on the prevention and treatment of various chronic diseases, including cancer, cardiovascular and neurodegenerative diseases, diabetes and atherosclerosis.^1^, ^2^, ^3^, ^4^ Among the phenolic compounds, anthocyanins stand out as water‐soluble pigments responsible for the reddish colouring of the pomegranate husk and arils, a key characteristic that determines the economic value of the fruit. Pomegranate husk colour ranges from white‐yellow to deep red and aril colour from light pink to very deep red depending on cultivar, reflecting not only the concentration of total anthocyanins, but also the anthocyanin profile.^5^, ^6^ In addition, anthocyanin biosynthesis in pomegranate is significantly influenced by cultural practices^7^ and environmental conditions, such as temperature, drought and salinity.^8^

Studies have shown that the temperature difference between day and night is a critical factor in stimulating anthocyanin biosynthesis in several fruit species, including grapes,^9^ pomegranate^10^ and blood orange,^11^ among others. However, the ‘Mollar de Elche’ pomegranate production area in Spain is facing severe challenges due to climate change, which has led to reduced rainfall, increased temperatures and higher solar radiation. These changes have reduced the daily thermal amplitude. Sustained increases in night‐time temperatures and more frequent heat waves negatively affect anthocyanin synthesis, resulting in reduced colour intensity in the skin and arils of the fruit. Previous research indicates that moderate daytime temperatures (20–25 °C) and cool nights are ideal for maximising the anthocyanin concentration in pomegranates,^10^ although another recent study claimed that low night temperature is the most determining factor for anthocyanin accumulation in pomegranate arils.^12^ The lack of these optimal climatic conditions leads to poor colour development, which is one of the most valued parameters of this fruit species in international markets.

Pomegranate, classified as a non‐climacteric fruit, requires careful handling during harvesting, transport and storage to preserve its quality and prolong its shelf‐life. Under ambient conditions, pomegranates have a limited shelf‐life, with high respiration rate and an accelerated senescence process. However, it is important to note that pomegranates are susceptible to chilling injury (CI) if stored at temperatures below 5 °C, which can affect their quality. For this reason, storage at temperatures between 7 and 10 °C, depending on cultivar, is recommended.^13^, ^14^ CI manifests itself mainly on the surface of the fruit with symptoms such as drying, browning and the appearance of depressions or pits. In addition, browning affects the internal skin surface and CI increases the susceptibility of the fruit to fungal infections.^13^, ^14^, ^15^ Previous reports have evidenced the usefulness of elicitors, applied as pre‐ or postharvest treatments, as appropriate tools to reduce CI and maintain pomegranate fruit quality traits during storage.^16^ Thus, postharvest dipping application of salicylic acid^17^ at 2 mmol L^−1^, oxalic acid^18^ at 2, 4 and 6 mmol L^−1^ and acetyl salicylic acid^19^ at 0.1, 0.5 and 1 mmol L^−1^ showed a significant reduction in CI symptoms, electrolyte leakage (EL) and lipid peroxidation by maintaining high levels of antioxidants and unsaturated fatty acids. In addition, Lorente‐Mento *et al*.^20^ indicated that postharvest 1‐methylcyclopropene and methyl jasmonate (MeJA), applied alone or as combined treatments, reduced CI, improved fruit firmness, increased antioxidant enzymes and total phenolics and reduced surface discolouration in pomegranates. On the other hand, preharvest application of MeJA^21^ at 1 and 2 mmol L^−1^ and combined pre‐ and postharvest application of MeJA^15^ at 5 mmol L^−1^ reduced CI by increasing the ratio of unsaturated to saturated fatty acids, inducing the content of bioactive compounds and preserving the activity of antioxidant enzymes.

Polyamines (PAs) are nitrogenous compounds of low molecular weight and polycationic nature and are present in all living organisms. In plants, the major polyamines are putrescine (PUT; 1,4‐diaminobutane), spermidine (SPD; *N*‐3‐aminopropyl‐1,4‐diaminobutane) and spermine (SPM; bis(*N*‐3‐aminopropyl)‐1,4‐diaminobutane). They are synthesised from the amino acid arginine and are widely considered as phytohormones or plant growth regulators.^22^ Despite being found in higher concentrations than typical plant hormones, they play a crucial role in a wide range of growth and developmental processes, such as cell division and differentiation, embryogenesis, cellular homeostasis, dormancy breaking, germination, flower bud development and fruit set, growth and ripening.^23^, ^24^ Furthermore, PAs are also involved in the synthesis of secondary metabolites by serving as precursors of other molecules related to response against biotic and abiotic stresses.^25^, ^26^, ^27^ On the other hand, previous studies have shown that postharvest application of PAs is an effective tool to mitigate the effects of CI in several fruit species.^16^, ^28^ Specifically, in ‘Wonderful’ pomegranate, PUT dipping treatments, at 2 and 3 mmol L^−1^ concentrations, reduced CI incidence during long‐term storage,^29^ as well as 1 mmol L^−1^ PUT on ‘Mridula’ pomegranate^30^ and 1 mmol L^−1^ PUT or SPD dipping or vacuum infiltrated treatments in ‘Mollar de Elche’,^31^ and these effects were attributed to maintenance of cell membrane integrity. However, despite this evidence regarding postharvest applications, no previous literature is available about the effects of preharvest applications of PAs on quality attributes of pomegranate fruit either at harvest or during postharvest storage.

Thus, in the context of sustainable agriculture, the study reported here aimed to evaluate the potential use of PUT and SPD, as natural tools, applied to pomegranate trees during the fruit developmental cycle, to improve crop yield and fruit quality properties at harvest and to reduce CI during cold storage at 2 °C (a chilling temperature for pomegranate), thereby prolonging the shelf‐life of the ‘Mollar de Elche’ cultivar.

## MATERIALS AND METHODS

### Plant material, preharvest treatments and postharvest experimental design

The study was carried out on pomegranate trees (‘Mollar de Elche’ cv.), grown on a commercial plot belonging to the company Hebegu SL, located in Albatera (southern Alicante, Spain), under similar climatic conditions and agricultural practices in 2022 and 2023. The 14‐year‐old trees were planted at 6 m × 5 m. Three blocks of three trees were randomly selected for each treatment, leaving one untreated tree between each block and one row of untreated trees between each row of treated blocks to avoid the border effect between treatments. The trees were sprayed with 2 L of aqueous solutions of SPD or PUT (Sigma‐Aldrich, Germany, purity ≥ 99%) at 0.01, 0.1 or 1 mmol L^−1^ in the 2022 season and at 0.01 or 0.1 mmol L^−1^ in 2023, containing 1 mL L^−1^ Tween 20 (Sigma‐Aldrich, Germany) as surfactant, and distilled water with 1 mL L^−1^ Tween 20 was used for the control trees. The treatments (made in different trees in 2022 and 2023) were applied by means of manual foliar spraying at four key moments of the fruit developmental cycle: (i) when the pomegranate fruit was 30% of its final size and before anthocyanin biosynthesis (26 and 30 June in 2022 and 2023, respectively), (ii) when the fruit is 50% of its final size (27 and 20 July in 2022 and 2023, start of the anthocyanin biosynthesis), (iii) 1 month before the commercial harvest (30 and 29 August in 2022 and 2023) and (iv) 4 days before harvesting (30 September in both years). The concentrations used in 2023 were based on results obtained in 2022, in which SPD and PUT at concentrations of 0.01 and 0.1 mmol L^−1^ were the most effective in increasing crop yield and fruit quality properties, such as external and internal colour and aril total phenolic and anthocyanin content at time of harvest. The application times of the treatments were based on a previous study by García‐Pastor *et al*.^32^ Once the fruits had reached the ripening stage according to the company's commercial criteria, based on fruit size (*ca* 90 mm in diameter), husk light‐red colour and total soluble solids content (*ca* 150 g L^−1^), they were harvested. In accordance with the specified criteria and due to the heterogeneity of the fruit with respect to its ripening process, two harvest dates were established: 4 and 20 October in 2022 and 4 and 19 October in 2023. On both harvest dates, the yield of each tree was quantified in terms of kilograms per tree and number of fruits per tree. The fruits of the initial harvest were used to evaluate the quality parameters at harvest in both seasons, as well as for the storage experiment in 2023 as follows. Ten homogeneous pomegranate fruits without visual defects (sunburn, cracking or husk mechanical damages) were randomly selected per tree, resulting in a representative harvest sample of 30 fruits per replicate. The pomegranates were immediately transported to the laboratory within an hour. Once in the laboratory, six lots of five fruits were performed at random for each replicate. One lot of each replicate was used to measure fruit properties at harvest (day 0), and the remaining ones were stored at 2 °C and a relative humidity (RH) of 85–90%. Then, after 30, 60 and 90 days of cold storage, one lot of each replicate (three lots per treatment; *n* = 3) was taken at random and stored for 2 days at 20 °C (55–60% RH) before the fruit properties, as detailed in the following, were measured.

### Weight loss and respiration rate

The weight loss was determined individually in each fruit and expressed as a percentage with respect to the initial fruit weight. The weight was measured using a digital balance (KERN 440‐35N, Balingen, Germany). The results were the mean ± SE. To measure respiration rate, five fruits of each replicate were placed in a 3 L plastic jar, which was then hermetically sealed for 1 h. Subsequently, 1 mL was withdrawn from the holder atmosphere and utilised for carbon dioxide (CO_2_) quantification. The analysis was performed using a Shimadzu TMGC‐2010 gas chromatography equipped with a thermal conductivity detector (GC‐TCD) working at 150 °C, while injector and oven temperatures were 110 and 50 °C, respectively, and the carrier gas was helium running at 50 mL min^−1^ as previously established.^31^ The results were expressed as mg CO_2_ kg^−1^ h^−1^ and are the mean ± SE.

### Fruit quality parameters

The firmness of each fruit was determined individually using a TA.XT2i model texturometer (Stable Microsystems, Godalming, UK). The texturometer was programmed to apply a pressure resulting in a deformation of 5% of the fruit equatorial diameter by using a coupled flat probe travelling at 2 mm s^−1^. Results were expressed as the ratio between the applied force and the achieved deformation (N mm^−1^) and are the mean ± SE. The external colour of the fruit was measured using a colourimeter (CR‐C200, Konica Minolta Camera Co., Kanto, Tokyo, Japan) based on the CIELAB colour coordinates (L*, a*, b*). For each fruit, three measurements were taken at equidistant points of the equatorial fruit perimeter, and the results were expressed in hue angle (arctan b*/a*). To evaluate aril colour, each fruit was cut through the equatorial plane and the colour of the arils was measured in triplicate in each one as described above. For both parameters, husk and aril colour, results are the mean ± SE of three replicates of five fruits (*n* = 3). Then, the arils of the five fruits from each replicate were taken and combined to obtain three biological samples (*n* = 3). Total soluble solids (TSS) content was measured in duplicate in the juice obtained by pressing and filtering through two cotton fabric layers *ca* 50 g of arils using a digital refractometer (PR‐101, Atago Co. Ltd, Tokyo, Japan). The results obtained as °Brix, equivalent to grams of sugars per 100 g were thereafter converted and expressed as g L^−1^ (mean ± SE). The measurement of the total acidity (TA) was carried out in duplicate by titration of 1 mL of juice diluted in 25 mL of distilled water with 0.1 mmol L^−1^ NaOH up to pH 8.1, by using an automatic titrator (785 DMP Titrino‐Metrohm AG, Herisau, Switzerland). Results were expressed as grams of malic acid equivalent per litre. The ripening index (RI) was calculated in duplicate for each replicate as the TSS/TA ratio, and the results were expressed as the mean ± SE of three replicates (*n* = 3).

### CI index and EL

The assessment of the CI in pomegranate fruit was carried out by visual analysis using a six‐level hedonic scale developed by García‐Pastor *et al*.,^15^ which allowed for a quantitative evaluation of both external and internal CI. The scale was based on the percentage of the external and internal husk area affected by CI symptoms, including dehydration, browning and pitting. The scale ranged from 0 to 5, with 0 for fruit with no CI symptoms, 1 representing 1–20%, 2 representing 21–40%, 3 representing 41–60%, 4 representing 61–80% and 5 representing more than 81% of the fruit external or internal husk surfaces showing CI symptoms. To quantify the CI index, the following formula was applied: CI index = Σ(scale level × number of fruits at that level)/total fruits evaluated). Results were expressed as the mean ± SE of three replicates (*n* = 3) of five fruits. To evaluate the EL, four discs (5 mm in diameter) were extracted from the husks of each of the five fruits per replicate, using a cork borer, and subsequently they were immersed in glass jars containing 25 mL of distilled water. The initial conductivity (IC) was determined after 3 h of constant agitation using an XS COND 51 conductivity meter (Scharlab, Barcelona, Spain). Thereafter, the discs were frozen overnight and then subjected to an autoclave treatment at 120 °C for 15 min, and cooled at room temperature before measuring the total conductivity (TC). The EL was calculated as the initial percentage of the total electrolyte content using the following formula: (IC/TC) × 100, as described by Lorente‐Mento *et al*.^33^

### Malondialdehyde (MDA)

The MDA content was assayed by homogenising 2.5 g of husk tissue with 10 mL of 10% trichloroacetic acid and then centrifuging at 10 000 × *g* for 20 min at 4 °C. Subsequently, a 2 mL aliquot of the supernatant was combined with 2 mL of 0.67% 2‐thiobarbituric acid and incubated at 90 °C for 20 min. Then, the mixture was rapidly cooled in an ice bath and centrifuged once more at 10 000 × *g* for a further 5 min at 4 °C. The absorbance of the resulting supernatant was determined at 450, 532 and 600 nm wavelengths using a UV‐2401PC spectrophotometer (Shimadzu Scientific Instruments, Columbia, MD, USA) and results were expressed in micromoles per kilogram in fresh way basis (μmol kg^−1^).^20^

### Total anthocyanin and total phenolic contents

Total anthocyanin content was quantified according to a previous report.^15^ Briefly, 5 g of arils was homogenised in 15 mL of a solvent mixture consisting of methanol, formic acid and water (80:1:19, v/v/v). The homogenate was centrifuged at 10 000 × *g* for 10 min at 4 °C, and the supernatant was collected. Total anthocyanin content was determined by measuring the absorbance of the supernatant at 520 nm using a UV‐2401PC spectrophotometer (Shimadzu Scientific Instruments, Columbia, MD, USA) and expressed as mg kg^−1^ of fresh weight of cyanidin 3‐*O*‐glucoside equivalents (Cy 3‐glu), using a molar absorption coefficient of 23 900 L cm^−1^ mol^−1^ and a molecular weight of 449.2 g mol^−1^. The results were the mean ± SE of three replicates.

Determination of total phenolic content was performed by homogenising 5 g of arils with 10 mL of methanol–water (80:20, v/v) containing 2 mmol L^−1^ NaF. The homogenate was centrifuged at 10 000 × *g* for 10 min at 4 °C, and the supernatant was used for subsequent total phenolic quantification using Folin–Ciocalteu reagent as a colorimetric assay.^15^ Briefly, 50 μL of extract was mixed with 2.5 mL of water‐diluted Folin–Ciocalteau (1:1, v/v), mixed and incubated for 2 min at room temperature. Then, 2 mL of sodium carbonate (75 g L^−1^) was added and the mixture was shaken and incubated in a water bath at 60 °C for 5 min. Thereafter, the absorbance was measured at 760 nm using a UV‐2401PC spectrophotometer (Shimadzu Scientific Instruments, Columbia, MD, USA). The results were expressed as milligrams of gallic acid equivalents (GAE) per kilogram of fresh weight. The measurements were recorded as mean ± SE of three replicates (*n* = 3).

### Statistical analysis

Data were statistically analysed using SPSS software (version 3.1 for Windows). Analysis of variance (ANOVA) was performed to assess the effects of treatments and storage time as sources of variation, ensuring a detailed evaluation of potential interactions between these factors (two‐way ANOVA). Mean comparisons were conducted using Tukey's test to identify significant differences among treatments at a significance level of *P* < 0.05 for each sampling date. Results were expressed as the mean ± SE of three replicates (*n* = 3), ensuring statistical reliability.

## RESULTS

### Effect of PA treatments on crop yield

In the 2022 experiment, PUT and SPD were applied at 0.01, 0.1 and 1 mmol L^−1^ concentrations and a significant increase in crop yield was observed for all treatments, except for 1 mmol L^−1^ PUT, which showed no significant difference from the control (supporting information, Fig. 1A). The highest effects, up to 30% of crop yield increase, were observed for 0.01 and 0.1 mmol L^−1^ SPD treatments. This increase was due to an increment in the number of fruits harvested per tree, which were *ca* 180 in control trees and as high as *ca* 242 and 248 in 0.01 and 0.1 mmol L^−1^ SPD‐treated trees, respectively (supporting information, Fig. 1B), The number of fruits per tree before applying the treatments were *ca* 260 in trees to be used as control and to be treated with PUT or SPD, and, in turn, *ca* 70% of developing fruit in control trees reached the commercial ripening stage, while 30% dropped due to environmental factors from the start of the experiment until harvest date. However, in treated fruit the percentage of harvested fruit was higher, reaching 95% in 0.1 mmol L^−1^ SPD‐treated trees. On the contrary, no significant effects of treatments with PAs were observed in fruit weight, which ranged from 310 to 345 g. According to these results, PUT and SPD at 0.01 and 0.1 mmol L^−1^ were selected to repeat the experiment in the 2023 season.

**Figure 1:**
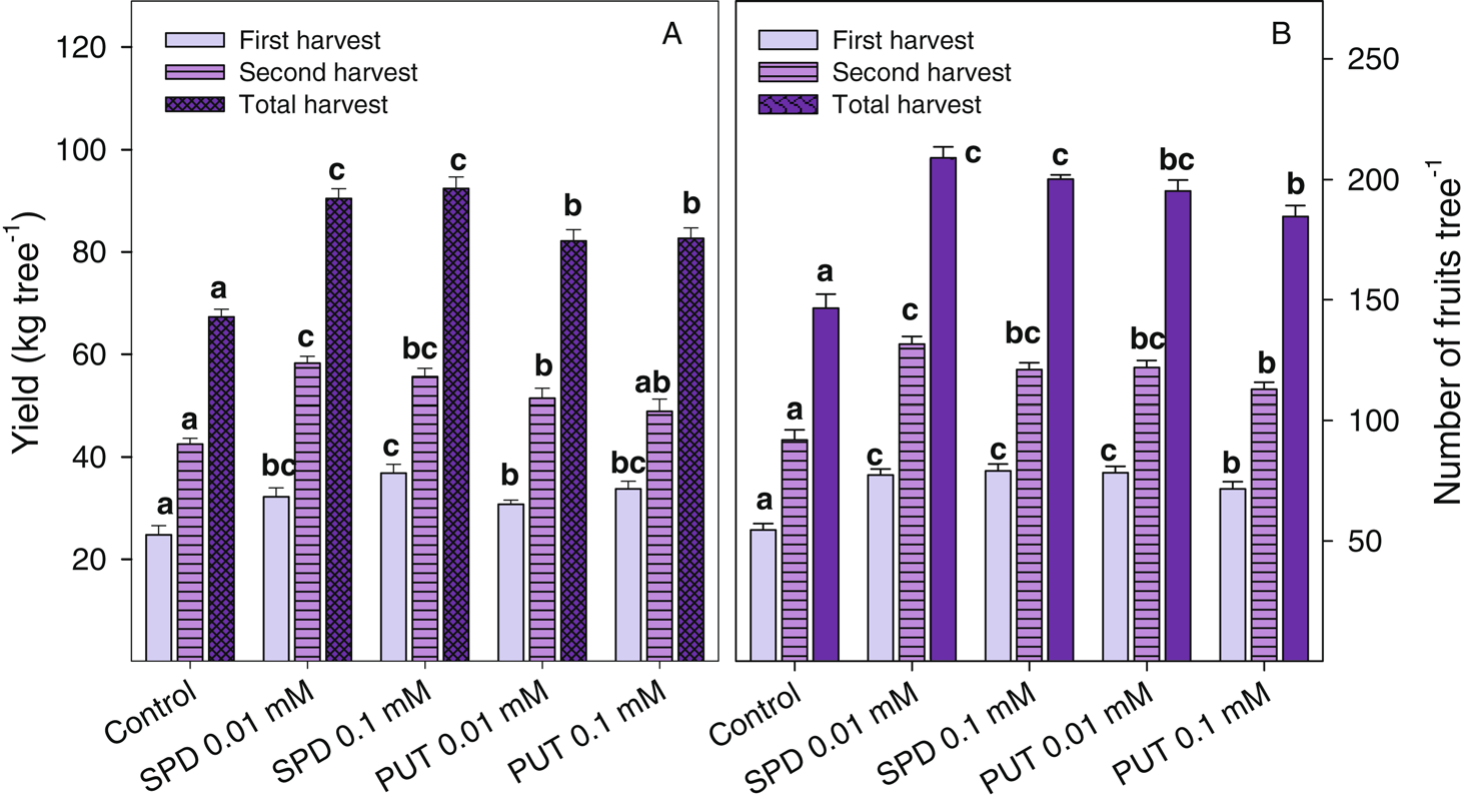
Yield at the first and second harvest and total yield (A) and number of fruits per tree at the first and second harvest and total number of harvested fruits (B) of ‘Mollar de Elche’ pomegranate as affected by 0.01 and 0.1 mmol L^−1^ PUT and SPD treatments. Data are the mean ± SE of three replicates of three trees per treatment. Different letters indicate significant differences (*P* < 0.05) between treatments for each harvest date or for total production.

In 2023, the yield of pomegranate trees increased significantly (*P* < 0.05) as a consequence of PA treatments as compared with controls, either in the first or in the second harvest (Fig. 1(A)). The highest yields were obtained with the application of SPD at concentrations of 0.01 and 0.1 mmol L^−1^, reaching 32.21 ± 1.76 and 36.82 ± 1.74 kg tree^−1^, respectively, in the first harvest, while in the second harvest, yields were 58.25 ± 1.37 kg tree^−1^ for SPD at 0.01 mmol L^−1^ and 55.61 ± 1.63 kg tree^−1^ for SPD at 0.1 mmol L^−1^. Regarding the total yield, values of 90.46 ± 1.92 kg tree^−1^ for 0.01 mmol L^−1^ SPD and 92.43 ± 2.23 kg tree^−1^ for 0.1 mmol L^−1^ SPD were obtained, which were *ca* 35% higher than that of control trees, confirming the results of the 2022 experiment. The total yields of PUT at 0.01 and 0.1 mmol L^−1^ were 82.16 ± 2.20 and 82.68 ± 2.04 kg tree^−1^, respectively, showing a 22% increase with respect to control trees (Fig. 1(A)).

A similar trend was observed in the total number of fruits harvested per tree, that is to say, a significantly higher total number of fruits per tree in the first and second harvest for all PA‐treated trees than for controls (*P* < 0.05). The treatment that resulted in the most significant increase in total fruit production was SPD at 0.01 and 0.1 mmol L^−1^, with 209.00 ± 4.61 and 200.11 ± 1.85 fruits per tree, respectively, while 146.56 ± 8.09 fruits per tree were harvested from controls (Fig. 1(B)). Given the fact that the initial number of fruits per tree was *ca* 215, 68% of the fruit developed until harvest in control trees, this percentage being increased by PA treatments, the highest percentages, 97% and 93%, being recorded for 0.01 and 0.1 mmol L^−1^ SPD, respectively. Fruit weight was not affected by PA treatments, ranging from 433 to 460 g, in agreement with results of the previous season.

### Respiration rate, weight loss and fruit quality parameters

Preharvest foliar application of PUT and SPD at concentrations of 0.01 and 0.1 mmol L^−1^ significantly reduced fruit respiration rate at harvest (*P* < 0.05) compared to controls, in both seasons (Table 1 and supporting information, Table S1), with no significant differences between PA treatments. During storage, the same trend was observed, although PUT treatments led to a higher reduction in the respiration rate than SPD treatments, with the most significant effect being observed in fruit treated with 0.01 mmol L^−1^ PUT at the last sampling date (Table 1). PAs significantly reduced weight loss during storage compared to the control. After 90 days of cold storage plus 2 days at 20 °C, fruit from control trees showed a weight loss of 20.95 ± 0.67%, which was significantly higher than that observed in fruit from all PA‐treated trees (Table 1). However, it is worth mentioning that most of the weight loses occurred during the additional two‐day shelf‐life period at 20 °C after the cold storage (data not shown). In terms of physicochemical parameters, PA treatments significantly improved fruit firmness at harvest, except for 1 mmol L^−1^ dose in the 2022 experiment (Table 1 and supporting information, Table S1). In addition, PA treatments led to significantly higher levels of firmness than the control group during cold storage. Pomegranates from 0.01 mmol L^−1^ PUT‐treated trees retained the highest firmness at the end of the postharvest storage with 18.31 ± 0.49 N mm^−1^, while values of 13.35 ± 0.58 N mm^−1^ were found in controls (Table 1). TSS content ranged from 146 to 155 g L^−1^, without significant differences among treatments at harvest or during storage (Table 1 and supporting information, Table S1). For TA, no significant differences were observed between fruit from control and PA‐treated trees at harvest, with values of 2.8–3.0 g L^−1^ in both trials (Table 1 and supporting information, Table S1). However, the decreasing trend in TA observed in controls during storge was delayed in those pomegranate fruit harvested from PA‐treated trees, leading to higher TA levels in fruit from PA‐treated trees than in controls in most of the sampling dates during storage (Table 1). Thus, the RI increased during storage, although it was significantly lower (*P* < 0.05) in fruit from PA‐treated trees compared to controls, the lowest RI values being recorded from PUT treatment at 0.1 and 0.01 mmol L^−1^, followed by 0.01 mmol L^−1^ SPD (Table 1).

**Table 1:** Respiration rate (RR), weight loss, firmness, total soluble solids (TSS), titratable acidity (TA) and ripening index (RI) in ‘Mollar de Elche’ as affected by preharvest SPD and PUT treatments, at 0.01 and 0.1 mmol L^−1^, at harvest (day 0) and after 30, 60 and 90 days of storage at 2 °C + 2 days at 20 °C.

Parameter	Days	Treatments				
		Control	SPD 0.01 mmol L^−1^	SPD 0.1 mmol L^−1^	PUT 0.01 mmol L^−1^	PUT 0.1 mmol L^−1^
RR (mg CO_2_ kg^−1^ h^−1^)	0	11.28 ± 0.28^b^	10.17 ± 0.25^a^	10.19 ± 0.24^a^	9.82 ± 0.25^a^	10.11 ± 0.16^a^
	30	12.86 ± 0.17^c^	11.83 ± 0.24^b^	11.94 ± 0.14^b^	10.79 ± 0.27^a^	11.00 ± 0.25^a^
	60	14.24 ± 0.32^d^	13.21 ± 0.13^c^	13.40 ± 0.14^c^	12.01 ± 0.24^b^	12.69 ± 0.25^a^
	90	17.47 ± 0.13^d^	15.43 ± 0.18^c^	15.71 ± 0.20^c^	13.88 ± 0.38^a^	14.87 ± 0.06^b^
Weight loss (%)	0	–	–	–	–	–
	30	10.78 ± 0.52^b^	8.31 ± 0.41^a^	8.23 ± 0.43^a^	7.84 ± 0.40^a^	7.73 ± 0.26^a^
	60	15.91 ± 0.72^b^	14.17 ± 0.62^a^	13.35 ± 0.53^a^	13.16 ± 0.53^a^	14.02 ± 0.77^a^
	90	20.95 ± 0.67^b^	18.39 ± 0.44^a^	18.00 ± 0.62^a^	17.89 ± 0.47^a^	18.84 ± 0.55^a^
Firmness (N mm^−1^)	0	30.99 ± 0.70^a^	35.65 ± 0.74^c^	33.08 ± 0.87^b^	36.27 ± 0.64^c^	35.68 ± 0.94^c^
	30	18.61 ± 0.82^a^	21.65 ± 0.63^ab^	21.03 ± 0.72^ab^	23.19 ± 0.70^b^	22.57 ± 0.76^b^
	60	15.68 ± 0.73^a^	18.55 ± 0.62^b^	18.80 ± 0.33^b^	20.53 ± 0.67^c^	19.23 ± 0.65^bc^
	90	13.35 ± 0.58^a^	14.69 ± 0.48^b^	15.95 ± 0.52^bc^	18.31 ± 0.49^d^	16.65 ± 0.47^c^
TSS (g L^−1^)	0	152.3 ± 1.9^a^	152.7 ± 1.0^a^	151.3 ± 1.2^a^	149.2 ± 1.9^a^	150.5 ± 1.8^a^
	30	154.2 ± 1.4^b^	153.3 ± 1.5^ab^	153.4 ± 1.2^ab^	150.4 ± 1.4^a^	151.7 ± 1.5^a^
	60	155.8 ± 1.4^b^	153.0 ± 1.3^ab^	153.2 ± 1.4^ab^	150.0 ± 1.3^a^	153.0 ± 1.3^ab^
	90	156.0 ± 1.3^b^	153.8 ± 1.0^ab^	154.5 ± 1.2^ab^	152.3 ± 1.4^a^	153.7 ± 1.5^ab^
TA (g L^−1^)	0	2.81 ± 0.11^a^	2.89 ± 0.12^a^	2.87 ± 0.20^a^	2.86 ± 0.13^a^	2.95 ± 0.21^a^
	30	2.61 ± 0.13^a^	2.65 ± 0.10^a^	2.81 ± 0.14^b^	2.76 ± 0.21^ab^	2.78 ± 0.13^ab^
	60	2.40 ± 0.11^a^	2.59 ± 0.13^ab^	2.72 ± 0.10^b^	2.65 ± 0.11^b^	2.65 ± 0.10^b^
	90	2.34 ± 0.12^a^	2.54 ± 0.11^ab^	2.64 ± 0.11^b^	2.64 ± 0.12^b^	2.59 ± 0.11^ab^
RI	0	54.20 ± 1.01^b^	52.83 ± 1.20^ab^	52.72 ± 0.41^a^	52.17 ± 0.50^a^	51.06 ± 0.57^a^
	30	59.08 ± 0.49^c^	57.85 ± 0.60^b^	54.59 ± 0.61^a^	54.49 ± 0.92^a^	54.56 ± 0.84^a^
	60	64.90 ± 1.09^c^	59.07 ± 0.72^b^	56.32 ± 0.68^a^	56.60 ± 0.53^a^	57.74 ± 0.79^ab^
	90	66.66 ± 0.57^c^	60.50 ± 0.69^b^	58.52 ± 1.45^ab^	57.68 ± 0.96^a^	59.34 ± 1.47^a^

Data are for the 2023 experiment and are expressed as mean ± SE. Different letters show statistically significant differences among treatments for each sampling date (*P* < 0.05).

**Figure 2:**
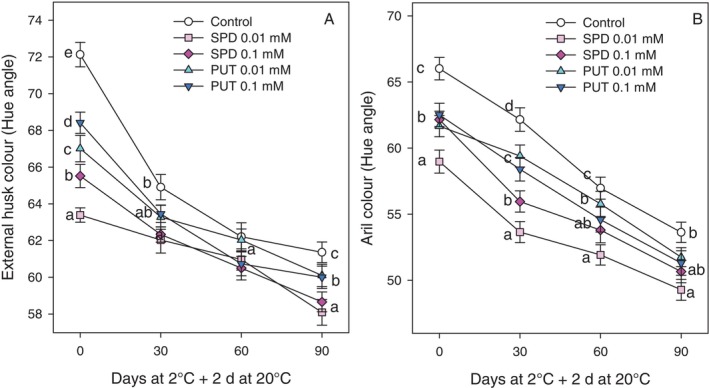
External husk colour (A) and colour of arils (hue angle) (B) of control and preharvest SPD‐ and PUT‐treated fruit at concentrations of 0.01 and 0.1 mmol L^−1^ of ‘Mollar de Elche’ pomegranate at harvest (0 days) and after 30, 60 and 90 days of storage at 2 °C + 2 days at 20 °C. The data are expressed as mean ± SE of three replicates, with each replicate consisting of five fruits collected from three trees per treatment. Different letters show statistically significant differences among treatments for each sampling date (*P* < 0.05).

The hue angle of fruit husk and arils was significantly lower (*P* < 0.05) in pomegranates from trees treated with PAs at 0.01 and 0.1 mmol L^−1^ doses than in controls at harvest in the 2022 and 2023 experiments (supporting information, Table S1; and Fig. 2). Decreases in hue values occurred during storage in all pomegranate fruit although values were lower in pomegranates from treated trees than in controls (Fig. 2). Thus, PA treatments increased red colour in husk and arils, since a lower hue angle means a deeper red colour. Furthermore, arils showed lower hue angle values than husk, indicating a deeper reddish tone in arils as compared with the external husk. Fruit treated with SPD at 0.01 mmol L^−1^ showed the lowest hue angle values as compared with the remaining treatments (Fig. 2). In these samples, the husk hue angle decreased from 63.39 ± 0.39 at harvest to 58.09 ± 0.70 after 90 days of cold storage plus 2 days at 20 °C (Fig. 2(A)), while hue angle in the arils ranged from 58.99 ± 0.87 at harvest to 49.28 ± 0.80 after the same storage period (Fig. 2(B)).

### CI, EL and MDA content

**Figure 3:**
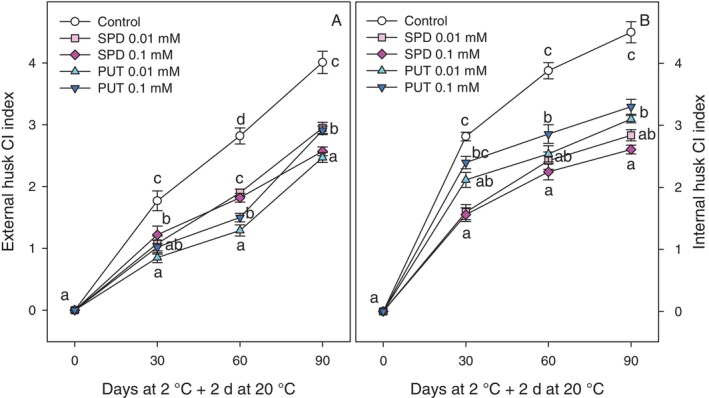
External (A) and internal (B) CI index of ‘Mollar de Elche’ pomegranate at harvest (0 days) and after 30, 60 and 90 days of storage at 2 °C + 2 days at 20 °C. Data are presented as mean ± SE of three replicates, each consisting of five fruits from three trees per treatment. Different letters indicate significant differences between treatments for each sampling date (*P* < 0.05).

Regarding the CI incidence, a linear increase in external CI index was observed in pomegranates during storage at 2 °C plus 2 days at 20 °C (Fig. 3(A)), while the most marked increase in internal CI occurred after the first month of storage (Fig. 3(B)). In general, the incidence of both external and internal CI symptoms was significantly higher in pomegranates from the control group (*P* < 0.05) compared to those from trees treated with PAs. Among treatments, the application of PUT at 0.01 mmol L^−1^ showed the greatest effectiveness in mitigating external husk CI incidence, reaching final CI index values of 2.67 ± 0.08, while for reducing internal husk CI index, the most effective treatments were SPD at 0.01 and 0.1 mmol L^−1^, with values of *ca* 2.75 (Fig. 3(B)). For control fruit, external and internal husk CI indexes at the last sampling date of storage were 4.01 ± 0.18 and 4.50 ± 0.17, respectively (Fig. 3(A),(B)).

**Figure 4:**
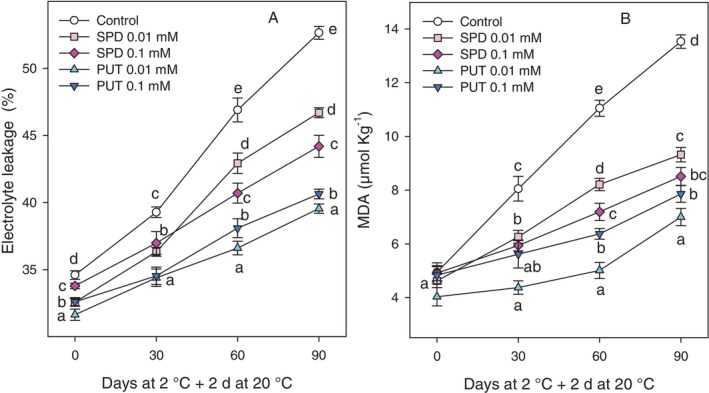
EL (A) and MDA content (B) in the husk of ‘Mollar de Elche’ pomegranate at harvest (0 days) and after 30, 60 and 90 days of storage at 2 °C + 2 days at 20 °C. Data are presented as mean ± SE of three replicates, each consisting of five fruits from three trees per treatment. Different letters indicate significant differences between treatments for each sampling date (*P* < 0.05).

At harvest, fruit from control trees exhibited significantly higher (*P* < 0.05) levels of EL compared to fruit from PA‐treated trees. During the cold storage period, EL increased progressively across all samples. However, fruit from all PA‐treated trees displayed a significantly lower EL, the lowest values being found for 0.01 and 0.1 mmol L^−1^ PUT treatments, with values of 39.55 ± 0.35% and 40.64 ± 0.36%, respectively, while values of 52.65 ± 0.48% were found in control fruit (Fig. 4(A)). The MDA content showed a similar trend to that of EL, with an increase over the postharvest storage and shelf‐life period. However, PA treatments led to significantly lower MDA levels compared to controls. After 90 days of cold storage plus 2 days at 20 °C, fruit treated with 0.01 and 0.1 mmol L^−1^ PUT had significantly (*P* < 0.05) lower MDA content with levels of 7.00 ± 0.32 and 7.86 ± 0.31 μmol kg^−1^, respectively, while that of the control group reached 13.53 ± 0.25 μmol kg^−1^ (Fig. 4(B)).

### Total anthocyanin and total phenolic contents

**Figure 5:**
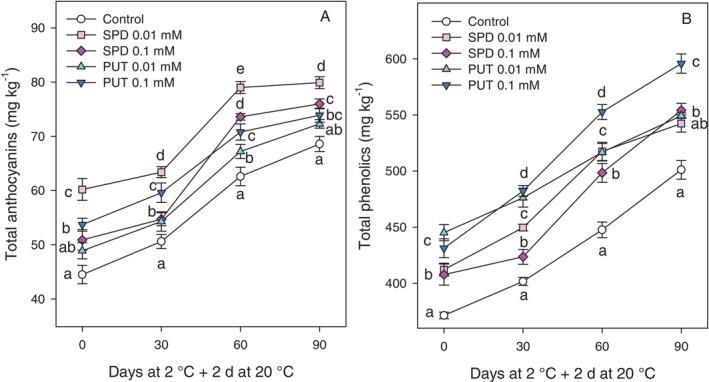
Effect of preharvest treatments with PAs on anthocyanin content (A) and total phenolic content (B) in the arils of ‘Mollar de Elche’ pomegranate at harvest (0 days) and after 30, 60 and 90 days of storage at 2 °C + 2 days at 20 °C. Data are presented as mean ± SE of three replicates, each consisting of five fruits from three trees per treatment. Different letters indicate significant differences between treatments for each sampling date (*P* < 0.05).

PA treatments led to significantly increased (*P* < 0.05) total anthocyanin content in the arils at harvest as compared to controls. The 0.01 mmol L^−1^ SPD treatment showed the highest effect, with levels of 72.20 ± 3.60 and 60.2 ± 1.2 mg kg^−1^ at harvest for 2022 and 2023, respectively, which were *ca* 35% higher than those in controls, for which values of 46.02 ± 2.07 and 44.5 ± 1.1 mg kg^−1^ for 2022 and 2023, respectively, were found (supporting information, Table S1; and Fig. 5(A)). An increasing trend was observed for anthocyanin concentration during the whole storage time, although enhanced levels were found for all PA treatments with respect to controls, reaching final values of 79.9 ± 3.1 mg kg^−1^ in arils from 0.01 mmol L^−1^ SPD treatment (Fig. 5(A)). With respect to phenolic content, it was also significantly enhanced by preharvest PA treatments at harvest in both years (supporting information, Table S1; and Fig. 5(B)), and although this parameter increased during cold storage in all samples, the differences between PA‐treated and control samples remained until the last sampling date (Fig. 5(B)). The strongest effect for this parameter during storage was found for 0.1 mmol L^−1^ PUT treatment, reaching values of 595.8 ± 8.6 mg kg^−1^ after 90 days of storage at 2 °C + 2 days at 20 °C, showing a 20% increase with respect to control fruit, for which values of 500.1 ± 8.3 mg kg^−1^ were observed (Fig. 5(B)).

## DISCUSSION

The southeast of Spain, which is the major production area of the ‘Mollar de Elche’ pomegranate, is experiencing the effects of climate change, including a decrease in precipitation, a persistent increase in temperature and an increase in solar radiation. This multifactorial combination of abiotic stresses negatively affects plant survival, plant growth and crop yield.^34^, ^35^ Recent studies highlight the crucial role of PAs in improving plant tolerance to different stressful conditions (salinity, drought and extreme temperatures), based on the results of exogenous PA applications or on the increase of endogenous levels of PAs under stress conditions.^24^, ^36^, ^37^, ^38^ In the present experiments, results indicated that the preharvest application of PAs played a crucial role in increasing the total crop yield, the major effects being observed for 0.01 and 0.1 mmol L^−1^ SPD treatments, *ca* 30–35% in 2022 and 2023 experiments, followed by 0.01 mmol L^−1^ PUT treatment, with *ca* 25% crop yield increase (Fig. 1 and supporting information, Fig. S1). This increase was directly related to the higher number of fruit harvested per tree, since no significant differences in individual fruit weight between the PA‐treated trees and controls were observed. In addition, 1 mmol L^−1^ PUT and SPD were also assayed in the 2022 experiment, and since no better effects were observed with respect to lower doses, this higher dose was discarded in the 2023 experiment, because lower doses would be more suitable in terms of cost‐effectiveness for practical applications. Therefore, similar effects were observed in both seasons, which could be only attributed to the effect of PA treatments since the register climatological data, such as mean monthly temperature and total monthly rainfall, were similar for both years (supporting information, Figs S2 and S3). It should be noted that the first PA treatments were applied after fruit set, when the fruit reached approximately 30% of their final size. Therefore, fruit set had occurred before treatment application and the effect of the treatments in increasing the number of fruits harvested per tree could be attributed to reduction of the natural fruit drop, due to wind or rain, that usually occurs during the rapid fruit growth and ripening periods. In fact, taking into account the number of fruits per tree at the start of the experiments, 70% and 68% of developing fruits were harvested from control trees in 2022 and 2023, respectively, and these percentages were increased in treated trees, up to 95% and 97% in 0.01 mmol L^−1^ SPD‐treated ones, respectively. In this sense, PAs have been reported to reduce shoot, flower and fruit drop by inhibiting the effect of ethylene on abscission, probably because ethylene and PAs compete for *S*‐adenosylmethionine, a common precursor in the metabolic pathways of both plant growth regulators.^24^, ^36^ On the other hand, most of the previous literature about the effects of PAs on increasing crop yield is related to their role in overcoming the negative impact of biotic and abiotic stressful factors on crop performance, leading to increased yield.^27^, ^37^ For instance, 1 mmol L^−1^ PUT treatment increased growth and yield in wheat under normal and water stress conditions, overcoming the negative impact of stees.^39^ Similarly, 1.5 mmol L^−1^ SPD treatment at flowering stage in rice increased grain yield per plant and weight of 1000 grains under heat stress conditions due to enhanced net photosynthesis rate and stomatal conductance, which were reduced by heat stress and recovered to similar values to those found in plants under normal conditions after SPD treatment.^40^ Moreover, increases in crop yield have also been reported in fleshy fruit species, such as apple,^41^ mango^42^ and sweet cherry,^38^ under commercial growth conditions as a consequence of PUT or SPD foliar tree treatments. These effects were attributed to an enhanced leaf area and chlorophyll content, ultimately increasing the net photosynthesis rate, showing that PAs could be an effective tool for improving agricultural productivity.

**Figure 6:**
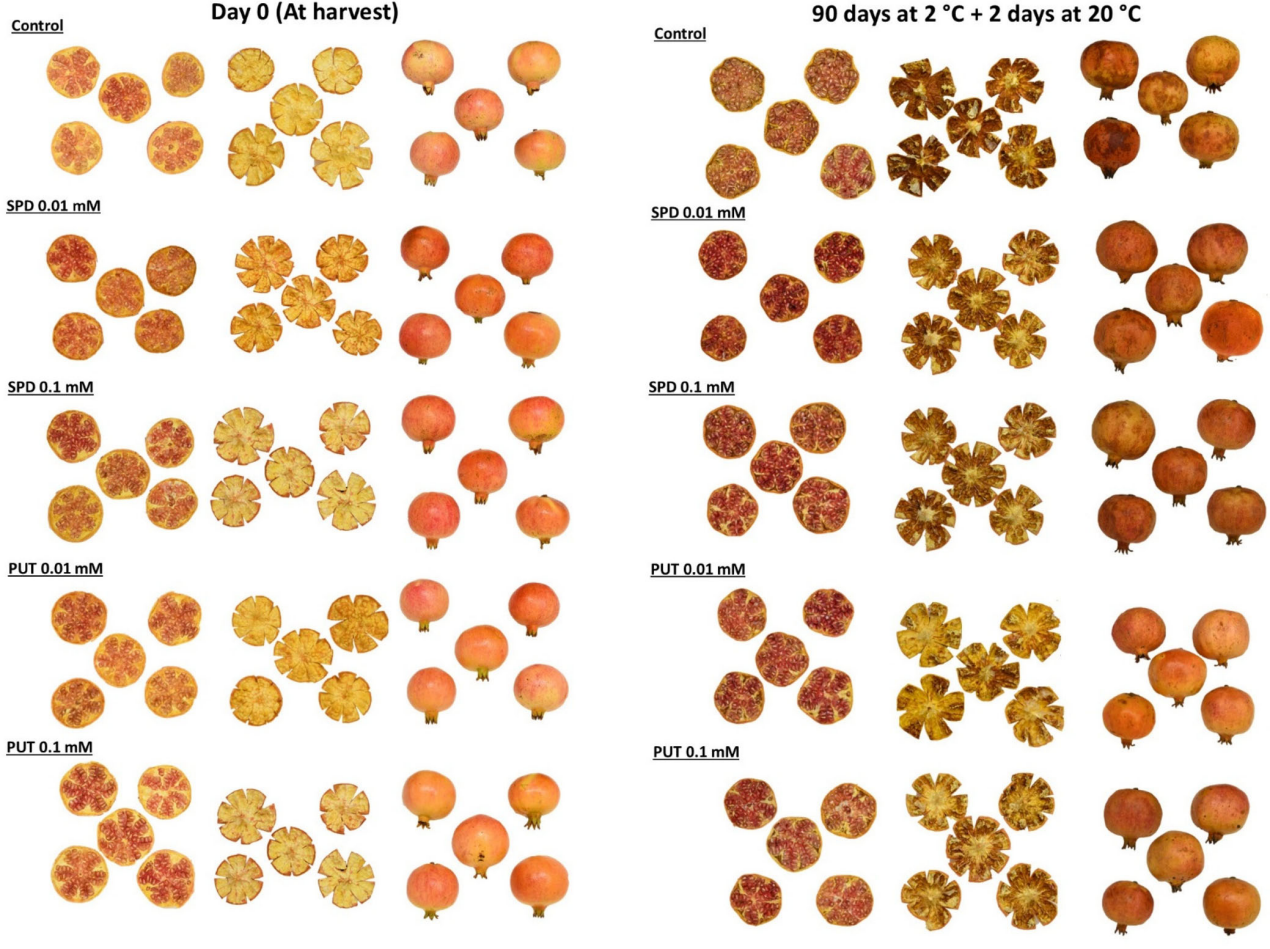
Photographs showing visual appearance of pomegranate arils and internal and external husk in fruit from control and PUT‐ and SPD‐treated trees at harvest and after 90 days of storage at 2 °C + 2 days at 20 °C.

With respect to pomegranate fruit quality properties during storage, the occurrence of CI is one of the major problems when fruit are stored at suboptimal temperatures, as in the present experiment. CI symptoms appear when fruit are exposed to ambient conditions after cold storage and accelerate the senescence process, reducing fruit shelf‐life and consumer acceptance.^13^, ^14^, ^15^ External CI in pomegranate is characterised by symptoms such as browning, spots and wrinkling on the surface of the fruit, while internal CI, on the other hand, appears as browning of the internal husk surface, as can be observed in Fig. 6. CI index, in both external and internal husk surface, increased with did storage time, but significantly lower values were observed in fruit from PUT‐ and SPD‐treated trees with respect to controls (Fig. 3(A),(B)). It has been suggested that larger pomegranate fruits could be more resistant to CI than smaller ones.^13^, ^14^, ^16^ However, fruit size was not affected by treatments and, in turn, the lower CI indexes found in fruit from PA‐treated trees should be attributed to the effects of treatments. Prolonged exposure to chilling temperatures induces a transition in the state of the cell membrane from a fluid crystalline liquid state to a rigid solid gel, which decreases the selective permeability of the membrane and can be assessed by measuring EL.^43^ In addition, low temperatures promote the accumulation of reactive oxygen species (ROS), generating oxidative stress leading to phospholipid hydrolysis and peroxidation of unsaturated fatty acids, quantifiable through the accumulation of MDA, and due to the increased activity of lipid‐catabolising enzymes, such as phospholipase D, lipase and lipoxygenase.^44^ In the present study, the increase of EL and MDA content during storage was reduced by PA treatments (Fig. 4), suggesting that PUT and SPD led to maintenance of cell membrane integrity, with the highest effect being observed for 0.01 mmol L^−1^ PUT. In fact, high correlations were observed between EL and MDA content or between EL and external CI index, the latter being also correlated with internal CI index (supporting information, Fig. 4). The effects of postharvest treatments with PAs on reducing CI have been reported in a wide range of fruit, such as apricot,^45^ plum,^46^ mango,^47^ melon^44^ and even in pomegranate.^16^, ^30^, ^31^ The mechanisms involved in this role of PAs have been related to (i) the enhanced activity of antioxidant enzymes, such as superoxide dismutase (SOD), peroxidase (APX) and catalase (CAT), which would reduce the amount of ROS leading to decreased lipid membrane oxidation and CI symptoms; (ii) the interaction of PAs with membrane phospholipids protecting membranes from peroxidation; and (iii) the maintenance of high unsaturated/saturated fatty acids ratio during storage leading to maintenance of membrane fluidity.^14^, ^16^, ^28^, ^45^ However, no previous reports are available regarding the role of preharvest PA application in reducing CI in fruit during storage.

PA treatments also reduced fruit respiration rate as well as weight, firmness and TA losses during cold storage, while TSS was not affected, generally (Table 1), showing a delay in the maturation and senescence processes during storage, in agreement with previous reports in other freshly fruit species after postharvest PA treatments.^48^ The effects of PAs on reducing fruit weight loss could be explained by their role in maintaining ionic and osmotic homeostasis, throughout their interaction with plasma membrane phospholipids, which helps to maintain membrane stability^49^ and the formation of phosphatidic acid, a key regulator that affects K^+^ efflux, modulation of membrane trafficking proteins, cytoskeletal organisation and ion transport.^50^, ^51^ On the other hand, the higher firmness levels found in pomegranate fruit harvested from PA‐treated trees with respect to controls could be attributed to the ability of PAs to bind to pectin in the cell wall, reinforcing its structure.^52^ The polycationic charge of polyamines, SPM^4+^ > SPD^3+^ > PUT^2+^, has been associated with a selectivity gradient in their capability of binding to cell wall and membrane components.^52^, ^53^ Thus, preharvest treatments with SPD at 1 and 2 mmol L^−1^ were more effective than PUT at similar concentrations on reducing softening in two table grape cultivars.^54^ On the contrary, the present results showed a different pattern because fruit from PUT‐treated trees showed higher firmness values than those from SPD‐treated ones during storage. In this sense, postharvest PUT and SPD treatments showed similar effects on delaying softening of carambola fruit and reducing *β*‐galactosidase activity, the main enzyme responsible for pectin degradation in this fruit species.^55^ Thus, the two positive charges of PUT could be enough to achieve its crosstalk with pectin components of the cell wall, stabilising and reinforcing its structure and hindering the access of pectin‐degrading enzymes.

In ‘Mollar de Elche’ pomegranate, colour is a key indicator of ripeness and quality. The colour break in the fruit, which marks the transition from chloroplasts to chromoplasts, is a visual indicator of ripening for both climacteric and non‐climacteric fruit, associated with changes in sweetness, acidity, texture and colour, mainly regulated by ethylene in climacteric fruit and by ABA in non‐climacteric fruit.^56^, ^57^, ^58^ The present study found that treatment with PAs increased the reddish colouration in husk and arils at harvest in the 2022 and 2023 experiments (supporting information, Table S1; and Fig. 2(A),(B)) and during cold storage in the 2023 experiment, as can be observed in Fig. 6. This fact is of commercial importance, since ‘Mollar de Elche’ pomegranate is a poor red‐coloured cultivar and the increase in anthocyanin content would enhance its value in international markets. Anthocyanin accumulation in the arils increased during storage with higher levels being found in fruit from PA‐treated trees than in controls (Fig. 5(A)). The major anthocyanins in pomegranate arils are delphinidin 3,5‐diglucoside, delphinidin 3‐glucoside, cyanidin 3,5‐diglucoside, cyanidin 3‐glucoside, pelargonidin 3,5‐diglucoside and pelargonidin 3‐glucoside which increase with maturation and are found at different concentrations depending on cultivar.^5^, ^6^, ^32^, ^59^ PA treatment also increased phenolic content in the arils at harvest, by 10–20% depending on treatment, and despite the fact that phenolic concentration in the arils increased along storage, higher levels were observed in fruit from PA‐treated trees (Fig. 5(B)). These results are consistent with previous experiments showing that preharvest treatments with PUT increased the concentration of anthocyanins and other phenolic compounds in grape,^54^ pear,^60^ apricot,^61^ mango,^42^ strawberry^62^ and sweet cherries.^38^ Accordingly, postharvest treatments with PAs maintained higher levels of these bioactive compounds in blood orange,^63^ mango,^47^ plum,^46^ strawberry^64^ and even in pomegranate.^65^ In addition, postharvest 2 mmol L^−1^ PUT treatment of kiwifruit maintained higher levels of total phenolics and flavonoids, as well as fruit quality traits during storgae.^66^ These effects could be attributed to the role of PAs in scavenging ROS, increasing the activity of the antioxidant enzymes SOD, APX and CAT, and preventing the activity of oxidative enzymes, such as polyphenoloxidase,^16^, ^28^ leading to pigment protection from oxidative damage during cold storage.^45^, ^64^ In addition, the PUT treatment of mango fruit has been reported to increase the activity and the relative gene expression of phenylalanine ammonia lyase enzyme and other enzymes involved in the biosynthesis of phenolics, including flavonoids and anthocyanins.^67^

Climate change, through its impact on temperature fluctuations, directly affects the coloration of both husk and arils in pomegranates.^10^, ^12^ The absence of optimal climatic conditions leads to poor colour development, compromising one of the most valued quality attributes of this fruit species in international markets. In this context, use of PAs may represent a promising strategy to mitigate the negative effects of climate change on fruit coloration and to increase the overall pomegranate quality, as has been confirmed in the present study.

## CONCLUSIONS

Exogenous application of PUT and SPD was demonstrated to be an effective strategy to increase crop yield and mitigate CI in ‘Mollar de Elche’ pomegranate fruit during a prolonged storage at 2 °C, by maintaining cell membrane integrity, manifested as reduced MDA accumulation and EL increase. In addition, PA treatments led to improved fruit quality (firmness, TA, colour and reduced weight loss) and functional properties, by increasing the content of phenolic compounds, especially anthocyanins. Particularly, treatment with SPD at 0.01 mmol L^−1^ showed the best results in the increase of crop yield and reddish coloration in husk and arils, while PUT at 0.01 mmol L^−1^ was the most effective in reducing the symptoms of CI. Therefore, the use of PAs, especially SPD at 0.01 mmol L^−1^, could represent a sustainable tool to enhance pomegranate crop yield, alleviate the incidence of CI and preserve the quality of pomegranates during cold storage, with important benefits for growers, marketers and consumers. Nevertheless, more research is needed to find out if the applied doses would lead to similar effects in other climatic conditions, pomegranate cultivars or fruit species, as well as to verify the feasibility and applicability of the proposed methodology under commercial orchard conditions.

## CONFLICT OF INTEREST

The authors declare that the research was conducted in the absence of any commercial or financial relationships that could be construed as a potential conflict of interest.

## AUTHOR CONTRIBUTIONS

Conceptualisation, MS and DV; methodology, FG‐A, .P‐M, MEG‐P, FG and SC; investigation, all authors; writing – original draft, JP‐M; writing – review and editing, MS and DV; visualisation, all authors; supervision, MS and DV; project administration, MS and DV; funding acquisition, MS and DV. All authors have read and agreed to the published version of the manuscript.

## Supporting information


**Data S1.** Supporting Information.

## Data Availability

The data that support the findings of this study are available from the corresponding author upon reasonable request.
