# Supplementary material for: Polyamine treatments: an effective strategy to improve crop yield and fruit colour and to preserve quality of ‘Mollar de Elche’ pomegranate fruit during storage at chilling temperatures
Source: J Sci Food Agric. 2026 Feb 25;106(7):4101–12. doi: 10.1002/jsfa.70542 (PMC13067093; doi:10.1002/jsfa.70542)
Supplement: Supplementary file 1 — Data S1. Supporting Information. [file JSFA-106-4101-s001.docx]

**Supplementary Fig. 1.** Yield (kg tree^-1^) (**A**) and number of fruits tree^-1^ (**B**) of ‘Mollar de Elche’ pomegranate in 2022 experiment as affected by putrescine (PUT) and spermidine (SPD) treatments at 0.01, 0.1 and 1 mM. Data are the mean ± SE of three replicates of three trees per treatment. Different letters indicate significant differences (*p* < 0.05) between treatments.

**Supplementary Fig. 2.** Average monthly temperature at the climate station of Elche (Alicante, Spain), close to the experimental field, for the two experimental years. T1, T2, T3 and T4 show the dates of treatments. https://www.aemet.es/es/datos_abiertos/AEMET_OpenData

**Supplementary Fig. 3**. Total monthly precipitation at the climate station of Elche (Alicante, Spain), close to the experimental field, during the two experimental years. T1, T2, T3 and T4 show the dates of treatments. https://www.aemet.es/es/datos_abiertos/AEMET_OpenData

B

A

A

**Supplementary Fig. 4.** Correlations between internal and external husk chilling injury (CI) index (A) and between electrolyte leakage and internal husk chilling injury (CI) index or malondialdehyde (MDA) content (B), taking into account data of control and treated pomegranates for all storage sampling dates.

**Supplementary Table 1**. Respiration rate (RR), firmness (N mm^-1^), external husk colour (Hue angle), internal colour of arils (Hue angle), total soluble solids (TSS, g L^-1^), titratable acidity (TA, g L^-1^), ripening index (RI), total anthocyanins (mg kg^-1^) and total phenolics (mg kg^-1^) in 'Mollar de Elche' at harvest in control and pre-harvest treated-fruits with spermidine (SPD) and putrescine (PUT) at concentrations of 0.01, 0.1 and 1 mM in 2022 experiment.

| Parameter | Treatments | | | | | |  |  |
| --- | --- | --- | --- | --- | --- | --- | --- | --- |
|  | Control | SPD 0.01 mM | SPD 0.1 mM | SPD 1 mM | PUT 0.01 mM | | PUT 0.1 mM | PUT 1 mM |
| RR CO_2_ (mg kg^-1^ h^-1^) | 11.68 ± 0.24 ^b^ | 10.80 ± 0.23 ^a^ | 10.65 ± 0.27 ^a^ | 10.32 ± 0.23 ^a^ | | 10.36 ± 0.16 ^a^ | 10.08 ± 0.21 ^a^ | 10.66 ± 0.24 ^a^ |
| Firmness (N mm^-1^) | 29.03 ± 0.64 ^ab^ | 31.88 ± 0.56 ^d^ | 31.09 ± 0.57 ^bcd^ | 29.85 ± 0.48 ^abc^ | | 31.57 ± 0.57 ^cd^ | 32.21 ± 0.58 ^d^ | 28.62 ± 0.55 ^a^ |
| External husk colour  (Hue angle) | 69.10 ± 0.54 ^b^ | 65.47 ± 0.50 ^a^ | 65.30 ± 0.69 ^a^ | 69.97 ± 0.61 ^b^ | | 64.45 ± 0.62 ^a^ | 64.78 ± 0.69 ^a^ | 68.95 ± 0.71 ^b^ |
| Internal colour of arils (Hue angle) | 63.79 ± 0.84 ^c^ | 51.67 ± 1.56 ^ab^ | 48.92 ± 1.31 ^a^ | 60.41 ± 1.28 ^c^ | | 49.89 ± 0.79 ^a^ | 54.61 ± 1.23 ^b^ | 61.98 ± 0.82 ^c^ |
| TSS (g L^-1^) | 147.3 ± 1.2 ^a^ | 150.0 ± 1.20 ^a^ | 151.0 ± 1.20 ^a^ | 151.3 ± 1.20 ^a^ | | 148.2 ± 1.20 ^a^ | 147.3 ± 1.10 ^a^ | 148.5 ± 1.20 ^a^ |
| TA (g L^-1^) | 2.88 ± 0.10 ^a^ | 2.93 ± 0.10 ^a^ | 3.0 ± 0.10 ^a^ | 2.9 ± 0.10 ^a^ | | 2.86 ± 0.10 ^a^ | 2.93 ± 0.10 ^a^ | 2.93 ± 0.10 ^a^ |
| RI | 51.14 ± 0.38 ^a^ | 51.19 ± 0.43 ^a^ | 50.33 ± 0.99 ^a^ | 52.17 ± 0.87 ^a^ | | 51.82 ± 0.91 ^a^ | 50.27 ± 0.67 ^a^ | 50.68 ± 0.76 ^a^ |
| Total anthocyanins  (mg kg^-1^) | 46.02 ± 2.07 ^a^ | 72.20 ± 3.60 ^c^ | 70.00 ± 0.80 ^c^ | 54.20 ± 2.10 ^b^ | | 55.80 ± 2.50 ^b^ | 56.20 ± 2.40 ^b^ | 53.30 ± 2.50 ^b^ |
| Total phenolics  (mg kg^-1^) | 348.8 ± 10.0 ^a^ | 436.65 ± 7.40 ^c^ | 443.5 ± 9.75 ^c^ | 420.7 ± 7.45 ^bc^ | | 475.3 ± 9.25 ^d^ | 468.00 ± 9.40 ^cd^ | 395.50 ± 9.95 ^b^ |
